# Application of a comprehensive sound environment management program to reduce the incidence of delirium in a pediatric intensive care unit: a quasi-experimental study

**DOI:** 10.3389/fmed.2025.1721666

**Published:** 2025-12-02

**Authors:** Huangjia Song, Hanjun Huang, Bingxin Wang, Yuru Zhang, Wen Qian, Yaqin Hu, Yaya Xu, Xiaohua Ge, Lili Xu

**Affiliations:** 1 Department of Pediatric Critical Care Medicine, Xin Hua Hospital Affiliated to Shanghai Jiao Tong University School of Medicine, Shanghai, China; 2 Shanghai General Hospital, Shanghai, China; 3 Institute of Hospital Development Strategy, China Hospital Development Institute, Shanghai Jiao Tong University, Shanghai, China

**Keywords:** pediatric intensive care unit, pediatric delirium, sound environment management, white noise, noise control, stress response, nursing intervention

## Abstract

**Objective:**

To evaluate the effectiveness of a sound environment management program in preventing pediatric delirium (PD) in the pediatric intensive care unit (PICU), and to provide evidence for improving the quality of nursing care and reducing PD incidence.

**Methods:**

A sound environment management program was implemented and evaluated using a quasi-experimental design. Patients admitted to the PICU of a tertiary general hospital in Shanghai, China, were recruited by convenience sampling. A total of 354 patients were included and divided into three groups: 139 patients admitted from October 2022 to May 2023 served as the control group (Group A); 109 patients admitted from July to November 2023 received sound environment control (Group B); and 106 patients admitted from December 2023 to April 2024 received sound environment control combined with white noise (WN) intervention (Group C). The control group received routine nursing care, while the intervention groups received additional noise control measures or noise control combined with WN masking. Differences among the three groups were compared in terms of PD incidence, subtype distribution, cortisol level changes, and noise parameters.

**Results:**

The incidence of PD was 47.5% (66/139) in Group A, 36.7% (40/109) in Group B, and 31.1% (33/106) in Group C (*p* = 0.027). The proportion of PD days was 30.7% (190/619) in Group A, 23.6% (107/453) in Group B, and 23.1% (100/433) in Group C (*p* = 0.006). The incidence of hypoactive PD was 18.7, 11.9, and 6.6% in Groups A, B, and C, respectively (*p* = 0.019). The time effect (*p* < 0.001) and group-by-time interaction effect (*p* = 0.002) for cortisol levels across PICU days were both statistically significant. Among the 24-h acoustic parameters, significant differences were observed in maximum sound level (LAmax, *p* < 0.001) and minimum sound level (LAmin, *p* < 0.001).

**Conclusion:**

Implementation of a comprehensive sound environment management program in the PICU effectively reduced environmental noise and PD incidence while attenuating stress responses in critically ill children. The program demonstrated significant clinical potential in improving nursing quality and promoting both psychological and physiological stability in pediatric patients.

## 1 Introduction

The Pediatric Intensive Care Unit (PICU) is an essential department for managing critically ill children; nevertheless, its technology-dependent setting introduces inherent complexities in patient care. While life-support equipment operates continuously, it inevitably generates a complex acoustic landscape—comprising sharp monitor alarms, rhythmic ventilator sounds, and the urgent conversations and operational noises of healthcare staff (1)—all of which create a persistently high-intensity noise environment (2, 3). Substantial evidence indicates that the average noise level in PICUs remains between 60 and 70 decibels (dB), a value that far exceeds the safe thresholds recommended by the World Health Organization (WHO) for hospital wards (35 dB during the day and 40 dB at night) (4). Such chronic noise exposure exerts profound physiological and psychological impacts on vulnerable pediatric patients (5). It has been demonstrated that noise is not merely an auditory burden but a potent non-specific stressor that disrupts sleep architecture and activates the hypothalamic–pituitary–adrenal (HPA) axis (6), subsequently leading to adverse clinical outcomes such as blood pressure fluctuations, decreased oxygenation, and elevated inflammatory responses (7).

Among these outcomes, pediatric delirium (PD)—an acute and fluctuating disturbance in attention and awareness—has received growing attention. Epidemiological data reveal that the prevalence of PD in PICU settings ranges from 12 to 47%, and may surge to 53–74% among mechanically ventilated children (8). Delirium is not a benign or self-limiting confusion of consciousness; rather, it is strongly associated with increased mortality, prolonged mechanical ventilation, higher hospitalization costs, and long-term cognitive impairment after discharge, forming a core component of Post-Intensive Care Syndrome (PICS) (9). Given its substantial adverse effects, the prevention of delirium has become a key focus in PICU quality improvement initiatives. The 2022 Society of Critical Care Medicine (SCCM) clinical practice guidelines strongly recommend non-pharmacological interventions as the cornerstone of delirium prevention, particularly those targeting modifiable environmental factors through multimodal approaches (10).

Among these environmental factors, noise has been identified as a central, modifiable risk factor for delirium onset. Its pathophysiological mechanisms are intricate and intertwined: on one hand, noise disrupts the sleep–wake cycle—especially by reducing rapid eye movement (REM) sleep—thereby depriving the brain of critical periods for rest and repair (11); on the other hand, persistent noise activates the sympathetic nervous system and the HPA axis, elevating stress hormones such as cortisol, amplifying systemic neuroinflammation and oxidative stress, and ultimately compromising the integrity of the blood–brain barrier and synaptic transmission, thus creating a neurobiological substrate for delirium (12). Despite growing awareness of the importance of noise control, traditional interventions—such as alarm management and behavioral training—can only partially reduce baseline noise levels. They are largely ineffective in addressing unpredictable, transient noises inherent to the PICU environment, resulting in limited overall efficacy (13).

To overcome these limitations, white noise (WN)—a sound signal with uniform energy distribution across all audible frequencies—has emerged as an innovative acoustic intervention strategy (14). The primary mechanism of WN is based on the auditory masking effect, whereby a stable, neutral, and continuous background sound masks or attenuates the perceptual salience of sudden, high-pitched noises (e.g., alarms, door slams), thereby reducing their arousal impact on the cerebral cortex (15). Preliminary studies have demonstrated that WN can improve subjective sleep quality, alleviate anxiety, and reduce agitation in adult ICUs, post-anesthesia care units, and neonatal intensive care units (16). However, systematic integration of WN into comprehensive PICU sound environment management programs, as well as rigorous evaluation of its efficacy in preventing PD—particularly the often-overlooked hypoactive subtype—remains scarce, with a lack of high-quality clinical trial evidence (17).

Therefore, this study aims to develop a structured PICU sound environment management protocol and evaluate its effectiveness in reducing the incidence and duration of PD, improving delirium subtypes and clinical outcomes. By analyzing the combined effects of noise reduction measures and WN intervention, this research seeks to provide scientific evidence to enhance the quality of nursing care for pediatric patients in intensive care and to minimize the occurrence of delirium.

## 2 Methods

### 2.1 Study design

A quasi-experimental design was employed to evaluate the effectiveness of the PICU sound environment management interventions.

### 2.2 Study setting and participants

Participants were recruited using a convenience sampling method from the PICU of a tertiary general hospital in Shanghai, China, between October 2022 and April 2024. The unit comprised 12 beds with a total area of approximately 320 square meters and featured an open-ward layout. During the study period, the average bed occupancy rate was about 82%. The daytime nurse-to-patient ratio was 1:2, and the nighttime ratio was 1:3. According to hospital regulations, parents were allowed to visit during designated hours but were not permitted to stay overnight.

Inclusion criteria: (1) age between 1 month and 18 years; (2) PICU stay of 24 h or longer; (3) informed consent obtained from the patient’s legal guardian.

Exclusion criteria: (1) patients in a persistent coma or deep sedation (Richmond Agitation-Sedation Scale (RASS) < −3) preventing PD assessment; (2) those diagnosed with severe psychiatric disorders (e.g., schizophrenia with significant behavioral or cognitive impairment); (3) patients with severe sensory dysfunction (complete blindness or deafness); (4) postoperative patients with irreversible neurological impairment following neurosurgery for severe traumatic brain injury; (5) those with preexisting central nervous system diseases (e.g., epilepsy, cerebral palsy, encephalitis); (6) patients with repeated or multiple PICU admissions.

Exclusion criteria: (1) withdrawal from the study upon guardian or patient request; (2) incomplete data records.

The required sample size was calculated using PASS 15.0 software, resulting in *n* = 288. Considering an estimated 10% attrition due to withdrawal or nonresponse, the final target sample size was set at 318, ensuring a minimum of 106 participants per group.

### 2.3 Grouping and interventions

**Figure 1:**
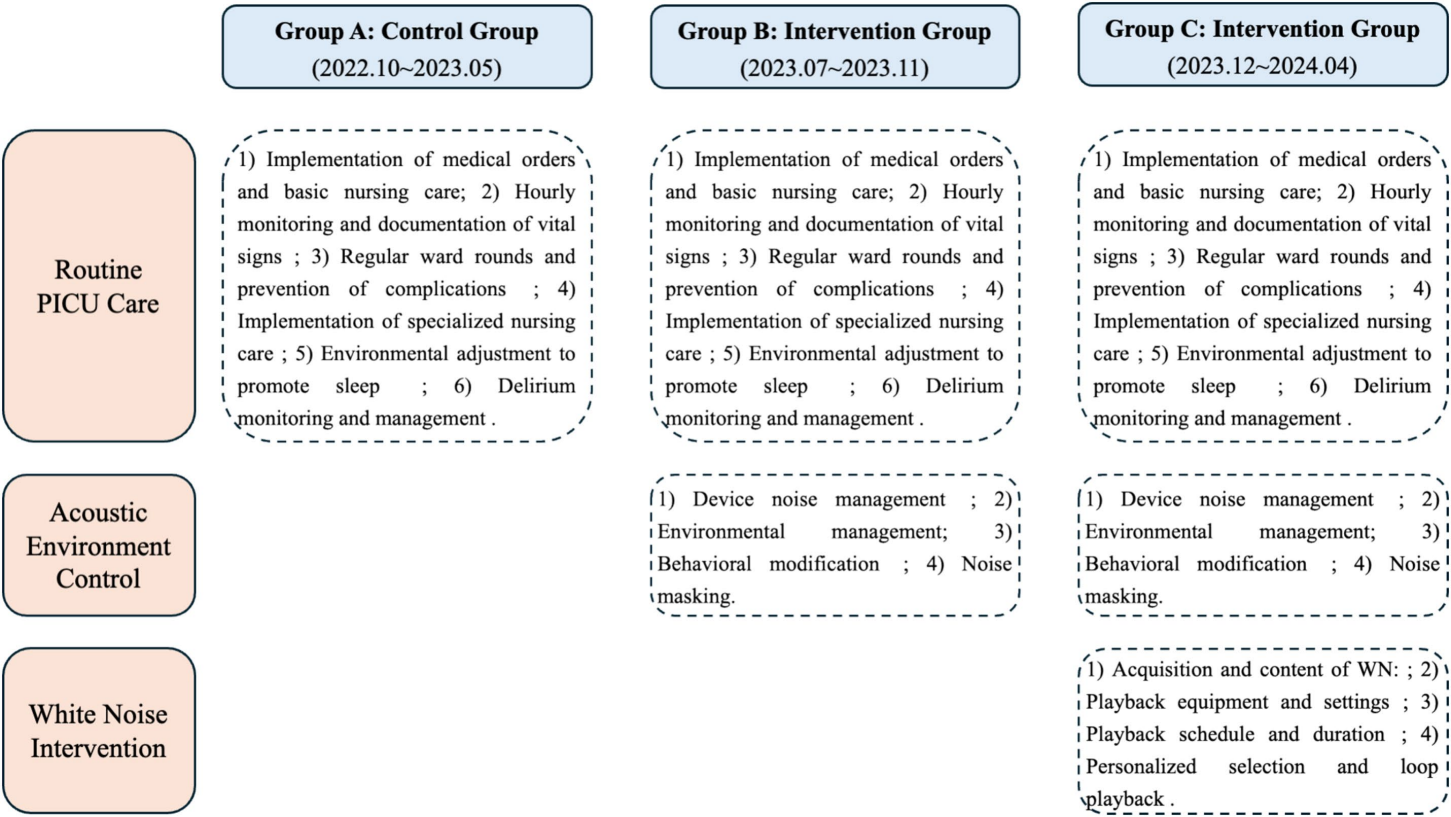
Flowchart of care interventions for the three PICU groups (routine care, acoustic environment control, and white noise intervention).

We divided PICU patients meeting admission criteria into three groups—A, B, and C—based on admission time. Group A served as the control group, while Groups B and C received interventions. Group A received routine care, Group B received routine care plus acoustic environment control, and Group C received routine care plus acoustic environment control plus white noise intervention (see Figure 1 for details). For specific intervention protocol information, please refer to the Supplementary material.

Based on the actual human resource situation within the department, an implementation team was established comprising one PICU associate chief physician, two PICU specialists, two PICU head nurses (one of whom is the researcher), and all responsible nurses who completed training and assessments. To ensure homogeneity of the intervention measures, we conducted standardized training for the intervention implementers. Through diverse and systematic training, dynamic assessments, and group discussions, we ensured the standardization of intervention measures and consistency in the evaluation system. The specific responsibilities of team members and the standardized training content are detailed in the Supplementary material.

### 2.4 Research instruments

#### 2.4.1 General information collection form

A structured questionnaire designed by the investigator was used to collect the following data:

①Demographic information: sex, age, and admission route (emergency or transfer);
②Disease-related information: diagnostic category, length of PICU stay, surgical history, disease severity, use of mechanical ventilation, physical restraint status, use of sedatives, and relevant laboratory indicators, including blood urea nitrogen, lactate, hemoglobin, and C-reactive protein. All laboratory values were collected from routine clinical tests performed within 24 h of PICU admission.

Diagnostic categories were classified according to the type of illness, including medical conditions, surgical conditions, and accidental injuries, to facilitate subsequent statistical analysis.

#### 2.4.2 Noise level in the PICU

**Figure 2:**
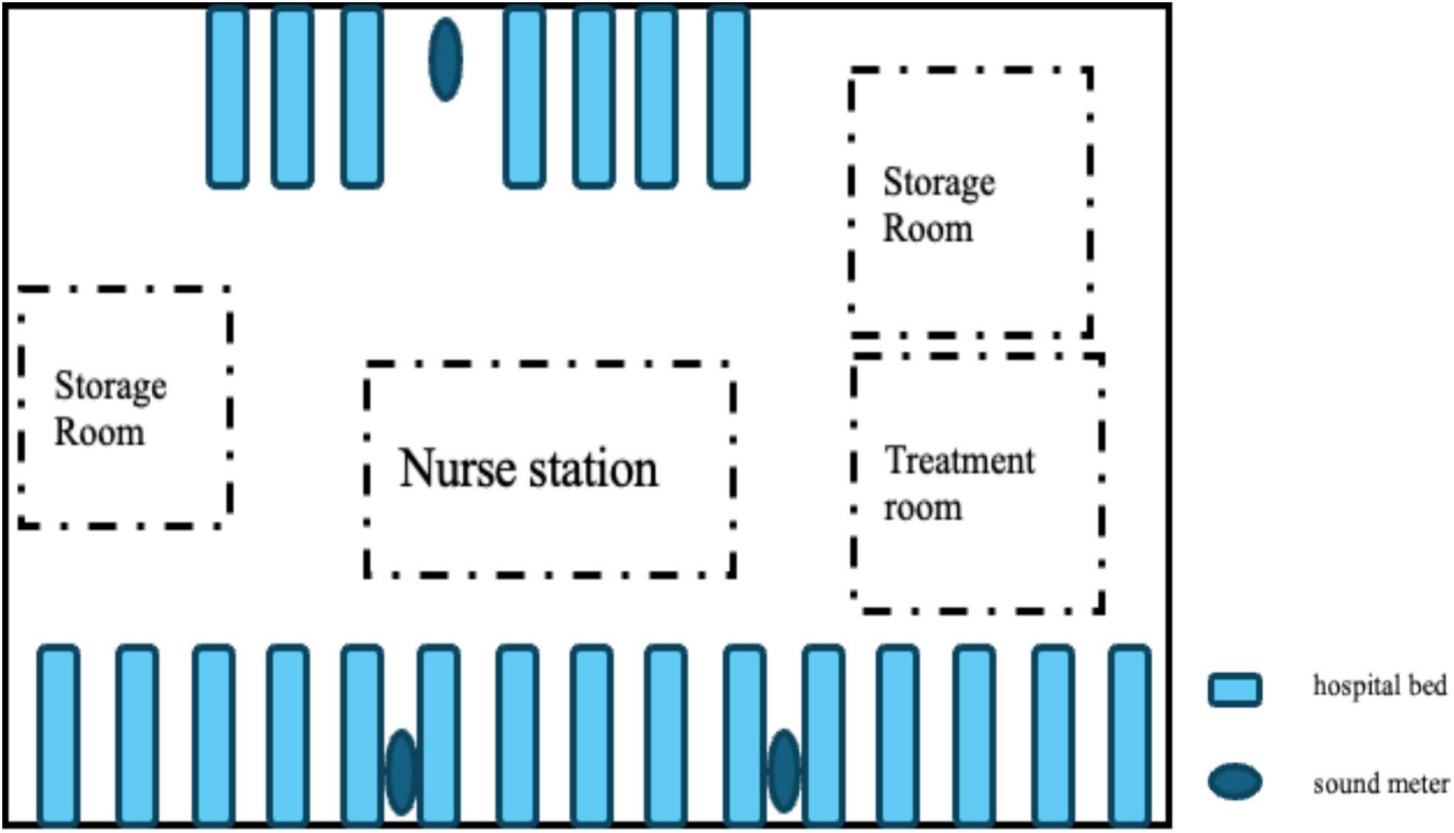
Schematic diagram of sound level meter placement.

The environmental noise level was measured using a Shengdawei high-precision sound level meter (model SW525B). The device automatically recorded A-weighted instantaneous sound levels every second, expressed in decibels (dB). Three sound meters were placed at designated positions within the unit: two on the side with more beds: the first located at one-third of the distance from the initial bed, and the second placed at the same proportional distance from the opposite end of the ward. One meter was placed on the side with fewer beds: placed at the midpoint of the bed row. Each meter was suspended on a fixed stand positioned 1.5 meters from the wall and 2 meters above the floor to ensure scientific validity and consistency of the monitoring points (Figure 2). The following acoustic parameters were recorded: LAmax (maximum sound level), LAmin (minimum sound level), LAeq (equivalent continuous sound levels), LAd (daytime equivalent sound level), and LAn (nighttime equivalent sound level).

#### 2.4.3 Assessment of PD

The Cornell Assessment of Pediatric Delirium (CAPD) was used to evaluate PD based on behavioral observation (18). It consists of eight items rated on a 5-point Likert scale, assessing awareness and cognitive function through observation of behavioral responses. When RASS ≥ − 3, the CAPD assessment was performed. A CAPD score greater than or equal to 9 indicated a positive diagnosis of PD. For children under two years of age, nurses referred to a developmental anchor chart to ensure age-appropriate interpretation of behaviors. Assessments were conducted twice daily at 08:00 and 20:00. These times were chosen to allow nurses sufficient time after shift handovers (07:00 and 19:00) to observe patient behavior and ensure accurate assessment.

#### 2.4.4 Assessment of sedation level

The RASS was used to assess sedation depth and classify PD motor subtypes (19). The scale ranges from +4 (combative) to −5 (deep sedation) across 10 levels. When RASS ≥ − 3, further PD assessment was conducted. Based on RASS scores, PD was categorized into three motor subtypes:

① Hypoactive PD: negative RASS scores;
② Hyperactive PD: positive RASS scores;
③ Mixed PD: fluctuating between positive and negative values and/or a score of 0.

#### 2.4.5 Assessment of comfort and pain

The Comfort Behavior Scale (CBS) was applied to evaluate sedation and analgesia levels through comprehensive behavioral observation of children under 18 years of age (20). It includes six behavioral indicators: alertness, calmness or agitation, respiratory response for ventilated patients (or crying for non-ventilated patients), body movement, muscle tone, and facial tension. Each item is rated from 1 to 5, with a total score ranging from 6 to 30. Higher scores indicate greater discomfort or pain severity.

#### 2.4.6 Assessment of disease severity

The Pediatric Risk of Mortality Score III (PRISM III), composed of 17 physiological indicators (21), was used to quantify disease severity and mortality risk. Parameters include systolic blood pressure, heart rate, body temperature, pupillary reflex, mental status, acidosis, total CO₂, pH, PaO₂, PaCO₂, glucose, potassium, creatinine, blood urea nitrogen, white blood cell count, prothrombin time, and platelet count. Each parameter is scored from 1 to 10 according to severity. Higher total scores indicate more severe illness and higher mortality risk. The PRISM III score was calculated based on data from the first 24 h of PICU admission.

#### 2.4.7 Assessment of functional status

The Functional Status Scale (FSS) was used to evaluate six functional domains: mental status, sensory function, communication, motor function, feeding, and respiratory status (22). Each domain is rated from 1 (normal) to 5 (very severe dysfunction), yielding a total score between 6 and 30. Higher scores indicate greater functional impairment. The change in FSS (ΔFSS) reflects functional variation during hospitalization. A ΔFSS ≥ 3 in total score or ≥ 2 in any domain was defined as new functional impairment, further classified as:

- Temporary impairment: new deterioration at ICU discharge that resolved by hospital discharge;
- Persistent impairment: deterioration present both at ICU and hospital discharge.

#### 2.4.8 Stress biomarkers

Cortisol secretion, a physiological response to stress, was measured to assess stress levels. Blood samples for cortisol measurement were collected between 08:00 and 09:00 on the 2nd day after admission (i.e., the morning of the first full day in PICU), with additional samples on days 3 and 7 if the patient remained admitted to the PICU. Samples were not collected after transfer out of the PICU. According to the laboratory reference standard, cortisol levels ≥ 625 nmol/L were considered abnormal.

#### 2.4.9 Unexpected events

Unexpected events included self-extubation attempts, accidental tube disconnection, catheter displacement, occurrence of pressure injuries, and the total number of such incidents during hospitalization.

### 2.5 Quality control

Rigorous quality control measures were implemented throughout the study. Prior to initiation, the research protocol was finalized through expert consultation. During the intervention phase, all data collectors received standardized training. Adherence to the intervention protocol and data collection procedures was monitored daily by the research team leads through direct observation and shift reports. To ensure data accuracy, a double-data-entry system was employed, with two independent teams entering data into separate databases for cross-verification. We affirm that no patient-identifiable information was shared via the WeChat group; it was used solely for reporting general adherence metrics. All statistical analyses were conducted under the supervision of a professional biostatistician.

### 2.6 Ethical considerations

This study adheres to the Declaration of Helsinki and was approved by the ethics committee of a tertiary general hospital in Shanghai (approval number: XHEC-C-2023-030-1). Authorization from the relevant departments was obtained prior to implementation. For all eligible patients, informed consent was acquired from their legal guardians.

### 2.7 Data collection

1. The responsible nurse assessed all eligible patients at least twice daily (typically at 08:00 and 20:00) using the RASS and CAPD to determine the presence and subtype of PD, and recorded the results. For children under two years, developmental anchor points were referenced (23). During each delirium assessment, sedation was evaluated with the CBS; both excessive and insufficient sedation were recorded as abnormal.
2. Baseline FSS assessment was completed by the investigator within 24 h of PICU admission, based on nursing records and guardian interviews reflecting pre-illness status. A follow-up FSS was obtained at PICU discharge. The difference between baseline and discharge scores was calculated to assess functional changes during hospitalization.
3. For biochemical monitoring, blood samples for cortisol measurement were collected on the morning of day 2 after admission and again on the morning of PICU discharge. For patients with prolonged hospitalization, additional samples were collected on days 3 and 7 if they remained in the PICU.
4. Noise monitoring data were exported every 24 h and saved in Excel files, including the maximum noise level and duration for each measurement. Given that the number of hospitalized patients and staff remained stable across the week, continuous noise data were recorded during the first week of each month throughout the study period.
5. General demographic and disease-related information were obtained from the electronic medical record system and used for data extraction.
6. The implementation of noise management measures was evaluated by the team leader, deputy leader, and head nurses using a participatory observation approach. Each participant was observed for three consecutive shifts (a total of 24 h). The findings were recorded in a self-designed “PICU Noise Management Intervention Implementation Evaluation Form.”

### 2.8 Statistical analysis

Data were analyzed using SPSS 25.0. Normally distributed continuous variables were expressed as mean ± standard deviation and compared between groups using the independent-samples *t* test. Non-normally distributed data were expressed as median (P25–P75) and compared using the Mann–Whitney U test; multi-group comparisons employed the Kruskal–Wallis H test. Categorical variables were expressed as frequencies and percentages and analyzed using the χ^2^ test or Fisher’s exact test. For repeated-measure data across multiple time points, generalized estimating equations were applied to assess group effects, time effects, and group-by-time interactions. All tests were two-tailed, and *p* < 0.05 was considered statistically significant.

## 3 Results

### 3.1 Comparison of general characteristics among the three groups

All participants were enrolled in strict accordance with the inclusion and exclusion criteria. From October 2022 to May 2023, 141 patients were included in the control group (Group A); from July to November 2023, 111 patients were included in the intervention group with environmental noise control (Group B); and from December 2023 to April 2024, 106 patients were included in the intervention group with environmental noise control combined with WN (Group C).

In Group A, two patients discontinued treatment on the second day of PICU admission due to disease deterioration. In Group B, one patient with severe pneumonia complicated by encephalitis and another who developed brain death during the intervention were excluded. Ultimately, a total of 354 pediatric patients were included in the final analysis: 139 in Group A, 109 in Group B, and 106 in Group C.

The basic demographic and clinical characteristics of the three patient groups are shown in Table 1.

**Table 1:** Comparison of general characteristics among the three groups of pediatric patients.

Variables	Group A (*n* = 139)	Group B (*n* = 109)	Group C (*n* = 106)	Test statistic	*P*
Sex [*n* (%)]					
Male	82 (59.0)	67 (61.5)	58 (54.7)	1.034a	0.596
Female	57 (41.0)	42 (38.5)	48 (45.3)		
Age [months, M (P25–P75)]	72.0 (12.0 ~ 132.0)	72.0 (24.0 ~ 120.0)	60.0 (23.75 ~ 108.0)	0.612b	0.736
Diagnostic category [*n* (%)]					
Medical diseases	77 (55.4)	76 (63.8)	67 (63.2)	8.724a	0.068
Surgical diseases	41 (29.5)	22 (20.2)	32 (30.2)		
Accidental injury	21 (15.1)	11 (10.1)	7 (6.6)		
Admission route [*n* (%)]					
Transfer	49 (35.3)	27 (24.8)	32 (30.2)	3.173a	0.205
Emergency	90 (64.7)	82 (75.2)	74 (69.8)		
Surgery [*n* (%)]					
Yes	52 (37.4)	29 (26.0.6)	28 (26.4)	4.706a	0.095
No	87 (62.6)	80 (73.4)	78 (73.6)		
Mechanical ventilation [*n* (%)]					
Yes	23 (16.5)	9 (8.3)	9 (8.5)	5.512a	0.064
No	116 (83.5)	100 (91.7)	97 (91.5)		
Physical restraint [*n* (%)]					
Yes	90 (64.7)	62 (56.9)	66 (62.3)	1.628a	0.443
No	49 (35.3)	47 (43.1)	40 (37.7)		
Use of sedatives [*n* (%)]					
Yes	23 (16.5)	21 (19.3)	21 (19.8)	0.514a	0.774
No	116 (83.5)	88 (80.7)	85 (80.2)		
RASS score [M (P25–P75)]	0.0 (0.0 ~ 1.0)	0.0 (0.0 ~ 1.0)	0.0 (0.0 ~ 1.0)	4.651b	0.098
PRISM III score [M (P25–P75)]	0.0 (0.0 ~ 3.0)	0.0 (0.0 ~ 2.5)	0.0 (0.0 ~ 0.0)	2.968b	0.227
Pain score [M (P25–P75)]	2.0 (1.0 ~ 2.0)	2.0 (2.0 ~ 2.0)	2.0 (1.0 ~ 2.0)	3.910b	0.142
Oxygenation index (mmHg) [M (P25–P75)]	433.3 (297.1 ~ 575.9)	395.2 (268.5 ~ 575.9)	433.3 (272.3 ~ 586.5)	0.838b	0.659
Length of PICU stay [M (P25–P75)]	4.0 (2.0 ~ 7.0)	4.0 (3.0 ~ 5.0)	4.0 (2.0 ~ 6.0)	2.433b	0.296
Duration of restraint [M (P25–P75)]	2.0 (0.0 ~ 5.0)	2.0 (0.0 ~ 4.5)	2.0 (0.0 ~ 4.0)	0.477b	0.788
Renal dysfunction [*n* (%)]					
Yes	23 (16.5)	24 (22.0)	19 (17.5)	1.257a	0.533
No	116 (83.5)	85 (88.0)	87 (82.5)		
Moderate to severe anemia [*n* (%)]					
Yes	65 (46.8)	42 (38.5)	38 (35.8)	3.346a	0.188
No	74 (53.2)	67 (61.5)	68 (64.2)		
Infection [*n* (%)]					
Yes	97 (69.9)	63 (57.8)	66 (62.3)	3.965a	0.138
No	42 (30.1)	46 (42.2)	40 (37.7)		
Blood urea nitrogen [M (P25–P75)]	5.0 (3.7 ~ 5.8)	4.4 (3.1 ~ 6.0)	4.2 (3.3 ~ 5.6)	0.508b	0.915
C-reactive protein [M (P25–P75)]	34.0 (8.0 ~ 111.0)	20.0 (8.0 ~ 69.5)	22.0 (5.0 ~ 99.0)	1.008b	0.438
Lactate [M (P25–P75)]	2.9 (2.1 ~ 3.9)	2.7 (2.1 ~ 3.7)	2.6 (1.8 ~ 3.4)	1.489b	0.482

PRISM III, Pediatric Risk of Mortality Score; (a) *χ^2^* test; (b) Kruskal–Wallis *H* test. Data are presented as median (interquartile range, P25–P75) for continuous variables or *n* (%) for categorical variables. Oxygenation index was measured on admission.

### 3.2 Monitoring of PICU noise levels

A 24-h monitoring study in the PICU revealed statistically significant intergroup differences in LAmax and LAmin (*p <* 0.001) when comparing changes in LAeq over 24 h among the three pediatric patient groups. Group A exhibited significantly higher LAmax and LAmin values than Groups B and C. Group A’s LAd was significantly higher than that of Groups B and C, with statistically significant differences among the three groups (*p* = 0.010). Detailed results are presented in Table 2.

**Table 2:** Comparison of 24-h equivalent continuous sound levels among the three groups.

Variables	Group A (*n* = 139)	Group B (*n* = 109)	Group C (*n* = 106)	*H*	*P*
LAmax	84.2 (83.2 ~ 85.4)	82.2 (80.5 ~ 82.8)#	81.8 (80.6 ~ 83.6)#	20.585	<0.001
LAmin	55.7 (54.8 ~ 56.0)	55.0 (53.94 ~ 55.3)#	54.5 (53.8 ~ 55.1)#	12.492	<0.001
LAeq	64.3 (63.0 ~ 66.0)	63.7 (61.5 ~ 65.1)	63.3 (62.3 ~ 64.6)	4.312	0.116
LAd	65.6 (64.4 ~ 66.5)	64.5 (63.7 ~ 65.3) #	64.3 (63.3 ~ 65.1)#	9.294	0.010
LAn	62.4 (60.4 ~ 63.3)	61.4 (59.6 ~ 61.9)	61.5 (60.5 ~ 62.7)	3.312	0.191

# indicates analysis performed using the Kruskal–Wallis *H* test; results show that compared with Group A, *p* < 0.05. Data are presented as median (interquartile range).

### 3.3 Comparison of daytime and nighttime noise levels in the PICU and differences from WHO standards

Across all three groups, the LAd was significantly higher than the LAn, with statistically significant differences (*p <* 0.001).

According to the recommendations of the Chinese Society of Critical Care Medicine (CSCCM), noise levels in ICUs should not exceed 45 dB during the daytime and 40 dB at night. The results showed that both LAd and LAn in the PICU markedly exceeded the CSCCM standards, with nighttime noise levels showing a particularly greater deviation from the recommended thresholds. Detailed results are presented in Table 3.

**Table 3:** Comparison of daytime and nighttime noise levels in the PICU and differences from CSCCM standard values.

Group	Daytime			Nighttime			*Z*	*P*
	Measured value (dB)	Standard value (dB)	Exceeding standard (%)	Measured value (dB)	Standard value (dB)	Exceeding standard (%)		
Group A	65.6 (64.4 ~ 66.5)	45	45.8	62.4 (60.4 ~ 63.3)	40	56.0	134.000	<0.001
Group B	64.5 (63.7 ~ 65.3)	45	43.3	61.4 (59.6 ~ 61.9)	40	53.5	135.000	<0.001
Group C	64.3 (63.3 ~ 65.1)	45	42.9	61.5 (60.5 ~ 62.7)	40	53.8	125.000	<0.001

# indicates analysis performed using the Kruskal–Wallis *H* test; results show that compared with Group A, *P* < 0.05. Data are presented as median (interquartile range).

### 3.4 Comparison of PD incidence and related indicators among the three groups

In Group A, 66 of 139 patients developed PD, with an incidence rate of 47.5%. In Group B, 40 of 109 patients developed PD (36.7%), and in Group C, 33 of 106 patients developed PD (31.1%). The overall difference in PD incidence among the three groups was statistically significant (*p* = 0.027). Further analysis revealed that the PD incidence in Group C was significantly lower than that in Group A (*p* = 0.010).

The distribution of PD motor subtypes also differed across the groups, particularly for hypoactive PD (*p* = 0.019). In Group A, hypoactive PD accounted for 39.4% of delirium cases, hyperactive PD for 33.3%, and mixed PD for 27.3%. In Group C, the proportion of hypoactive PD decreased to 21.2%, while hyperactive PD increased to 57.6%. The incidence of low-activity delirium in Group C was significantly lower than that in Group A (*p* = 0.006).

At PICU discharge, FSS scores differed significantly among the three groups (*p* = 0.010), with Group C showing greater improvement in functional status.

The incidence of new functional impairment was also significantly different: 18% in Group A, which was notably higher than in Groups B and C (*p* < 0.001).

Regarding unexpected events, no significant differences were found among the groups for individual events (*p* = 0.462, 0.436, 0.181, 0.744), whereas the total number of unexpected events differed significantly (*p* = 0.040). Detailed results are shown in Table 4.

**Table 4:** Comparison of PD incidence and related indicators among the three groups of pediatric patients.

Variables	Group A	Group B	Group C	*χ*^2^/*H*	*P*
PD cases [*n* (%)]	66 (47.5)	40 (36.7)	33 (31.1)#	3.632	0.027
Duration of PD (days)	2.0 (1.0 ~ 3.0)	2.0 (1.0 ~ 3.0)	2.0 (1.0 ~ 4.0)	0.918	0.632
Onset time of PD (days)	0.0 (0.0 ~ 1.0)	0.0 (0.0 ~ 1.0)	0.0 (0.0 ~ 1.0)	0.516	0.772
PD motor subtypes [*n* (%)]					
Hypoactive PD	26 (39.4)	13 (32.5)	7 (21.2)#	7.948	0.019
Mixed PD	18 (27.3)	13 (32.5)	7 (21.2)	2.761	0.251
Hyperactive PD	22 (33.3)	14 (35.0)	19 (57.6)	1.072	0.585
Number of patients with abnormal cortisol concentrations by length of hospital stay [*n* (%)]					
Day 1 of PICU stay	33 (23.7)	18 (16.5)	25 (23.6)	2.295a	0.318
Day 3 of PICU stay	14 (13.5)	7 (8.3)	6 (7.8)	2.017a	0.365
Day 7 of PICU stay	7 (15.6)	0 (0.0)	1 (9.1)	4.175b	0.100
FSS score					
At PICU admission	7.0 (6.0 ~ 10.0)	7.0 (6.5 ~ 9.0)	8.0 (6.0 ~ 10.0)	1.052	0.591
At PICU discharge	6.0 (6.0 ~ 9.0)	6.0 (6.0 ~ 7.0)	6.0 (6.0 ~ 7.25)#	9.293	0.010
Change in FSS (ΔFSS)					
New functional impairment [*n* (%)]	25 (18.0)	3 (2.8)#	2 (1.9)#	26.744	<0.001
Unexpected events [*n* (%)]					
Total number of unexpected events	138 (24.8)	82 (18.8)#	83 (19.5)	6.448	0.040
Self-extubation tendency	65 (46.7)	43 (39.4)	40 (37.7)	1.543	0.462
Accidental tube disconnection	33 (23.7)	18 (16.5)	23 (21.6)	1.659	0.436
Catheter displacement	37 (26.6)	20 (18.3)	18 (16.9)	3.416	0.181
Pressure injury	3 (2.1)	1 (0.9)	2 (1.8)	0.590	0.744

*n* indicates the number of pediatric patients. # indicates analysis performed using the Kruskal–Wallis *H* test; results show that compared with Group A, *P* < 0.05. (a) *χ^2^* test; (b) Fisher’s exact test. Data are presented as median (interquartile range) of FSS score, duration of PD and onset time of PD.

### 3.5 Comparison of PD days among the three groups

In Group A, the total PICU stay was 619 patient-days, with 190 PD days, accounting for 30.7% of the total stay.

In Group B, the total PICU stay was 453 patient-days, with 107 PD days (23.6%). In Group C, the total PICU stay was 433 patient-days, with 100 PD days (23.1%).

The comparison of PD days among the three groups showed statistically significant differences (*p* = 0.006), particularly in the number of hypoactive and hyperactive PD days (*p* = 0.001, 0.006). Detailed results are shown in Table 5.

**Table 5:** Comparison of PD days among the three groups of pediatric patients.

Variables	Group A (*n* = 619)	Group B (*n* = 453)	Group C (*n* = 433)	*χ* ^2^	*P*
PD days (*d*, %)	190 (30.7)	107 (23.6)#	100 (23.1)#	10.117	0.006
Hyperactive type (*n*, %)	77 (12.4)	28 (6.2)#	28 (6.5)#	16.958	0.001
Hypoactive type (*n*, %)	60 (9.7)	36 (7.9)	19 (4.4)#	10.246	0.006
Mixed type (*n*, %)	53 (8.6)	43 (9.5)	53 (12.2)	3.985	0.136

n indicates the number of PICU patient-days. # indicates analysis performed using the Kruskal–Wallis *H* test; results show that compared with Group A, *p* < 0.05.

### 3.6 Comparison of cortisol concentration changes at different PICU days among the three groups

Results from the Generalized Estimating Equation (GEE) analysis revealed significant intergroup differences, time effects, and group-by-time interaction effects in cortisol concentrations among the three groups. Both the time effect and the interaction effect were statistically significant (*P*time < 0.001, *P*interaction = 0.002), indicating that the impact of different interventions on cortisol levels varied significantly with increasing duration of PICU stay. The significant decline in cortisol levels was particularly evident on day 7 in Groups B and C, suggesting a cumulative beneficial effect of the interventions over time. Detailed results are presented in Table 6.

**Table 6:** Comparison of cortisol concentration changes at different PICU days among the three groups.

Group	Day 1 of PICU stay	Day 3 of PICU stay	Day 7 of PICU stay	Wald *χ*^2^ (Group)	Wald *χ*^2^ (Time)	Wald *χ*^2^ (Interaction)
Group A (*n* = 139)	321.9 (213.0 ~ 5,325)	293.5 (214.5 ~ 461.5)	325.4 (215.5 ~ 431.1)	5.990 (1)	116.221 (2)	16.992 (3)
Group B (*n* = 109)	328.7 (199.4 ~ 527.4)	252.0 (155.1 ~ 362.5)	198.3 (130.0 ~ 279.4)#			
Group C (*n* = 106)	374.5 (287.5 ~ 540.9)	251.7 (203.6 ~ 365.1)	203.7 (150.2 ~ 303.0)#			
*H*	4.865	5.968	14.749			
*P*	0.088	0.051	<0.001			

*p* values from GEE analysis: *P* (group) = 0.05, *P* (time) < 0.001, *P* (interaction) = 0.002. #indicates analysis performed using the Kruskal–Wallis *H* test; results show that compared with Group A, *P* < 0.05. Data are presented as median (interquartile range) of cortisol concentrations (nmol/L).

## 4 Discussion

### 4.1 Integrated effects of sound environment interventions on noise control and PD prevention in the PICU

Through dynamic monitoring of PD levels and functional status, this study provided a preliminary exploration of the potential physiological mechanisms underlying sound environment interventions. Although baseline PD levels in all groups remained within the normal reference range, the intervention groups showed an overall downward trend and a significant group-by-time interaction, suggesting that acoustic regulation may help promote a more favorable stress adaptation process during the PICU stay. It should be noted that the decline in PD may also be partly attributable to the natural recovery of critically ill children. Nevertheless, the concurrent decrease in PD levels and the incidence of pediatric delirium implies a possible association between environmental noise control and the attenuation of neuroendocrine stress responses. By reducing the frequency and amplitude of noise peaks, the intervention may have minimized unnecessary arousal stimuli, thereby helping patients maintain a more stable physiological state.

Previous ICU research in the United States has shown that average noise levels consistently exceed WHO-recommended thresholds (35 dB daytime, 40 dB nighttime), leading to overactivation of the HPA axis, abnormal cortisol secretion, and disrupted circadian rhythm, thereby increasing delirium risk (24). In this study, standardized alarm management, behavioral regulation, and real-time noise monitoring effectively reduced high-decibel peak events and improved acoustic stability. This process interrupted the pathological chain of “noise–arousal–stress–delirium,” alleviating neuroendocrine stress responses in children.

Although the 24-h LAeq did not differ significantly among the three groups, PD incidence declined notably, suggesting that temporal noise patterns rather than average intensity exert greater influence on central arousal. The unpredictability of sudden peaks is a key trigger for neural excitation and cognitive fluctuation. Bandyopadhyay et al. reported a linear correlation between peak noise frequency and delirium incidence, which aligns with our findings (25). The results suggest that flattening peak events and maintaining circadian continuity stabilize arousal and sleep–wake balance. The “peak-trimming and valley-filling” strategy may have smoothed the acoustic exposure curve, potentially improving sleep continuity and circadian synchronization, which could contribute to reduced delirium incidence. Similarly, Martinez et al. found that controlling peaks, rather than average sound levels, more effectively increased deep sleep proportion (26).

Through nurse-led behavioral and environmental regulation, this study achieved precise noise management, offering a low-cost, high-compliance, and scalable model for resource-limited PICUs. It also underscores the pivotal role of nursing teams in multidimensional environmental regulation and PD prevention in critical care.

### 4.2 Neural regulatory mechanisms of white noise masking and its advantages in preventing hypoactive PD

Building on routine sound environment management, this study introduced WN intervention for the first time to establish a dual-layer “noise control + auditory masking” framework. The incidence of PD in Group C decreased significantly from 47.5 to 31.1%, and hypoactive PD from 39.4 to 21.1%, indicating that WN substantially enhanced delirium prevention effectiveness. Hypoactive PD, often characterized by apathy and somnolence, is prone to underdiagnosis; its pathogenesis involves cholinergic inhibition and persistent neuroinflammatory activation (27). WN, as a spectrally uniform and non-directional auditory signal, masks unpredictable external stimuli, stabilizes arousal thresholds, and attenuates excessive thalamocortical excitation, thereby reducing neural hyperarousal (28).

Neurophysiological studies in Sweden have shown that WN exposure enhances *α*-wave activity while suppressing *β*-wave discharge, keeping the brain in an “alert resting” state that stabilizes attention and emotional regulation (29). Further fMRI research confirmed that WN modulates coupling between the Default Mode Network (DMN) and Salience Network (SN), improving sensory prediction consistency and reducing cognitive interference from abrupt stimuli (30). The present findings align with these mechanisms. Continuous auditory background provided by WN offered a “sensory shielding” effect for children in noisy environments, preventing transient hyperactivation of the auditory cortex and amygdala (31). This ‘auditory stabilization’ may have indirectly improved sleep quality, reduced cortisol fluctuations, and stabilized heart rate variability, thereby potentially disrupting the neuroendocrine cascade leading to PD. (28, 31)

Beyond physiological regulation, WN exerted psychological benefits. Environmental noise unpredictability elevates vigilance and anxiety, whereas a consistent auditory background enhances safety perception and sense of control, attenuating stress activation (32). Riedel et al. demonstrated that WN significantly prolonged deep sleep and reduced agitation episodes (33). The present findings are consistent with a similar ‘psychological soothing–physiological stabilization’ mechanism observed in prior studies of sensory modulation (30, 32). It can be inferred that WN acts through dual pathways: externally masking harsh acoustic stimuli and internally stabilizing neural rhythms, jointly suppressing delirium risk. This finding transcends the limitations of traditional physical noise reduction and highlights the potential of sound environment management in neuropsychological nursing.

### 4.3 Physiological stress modulation and neural recovery mechanisms of sound environment interventions

Dynamic monitoring of cortisol levels and functional status revealed the physiological mechanisms underlying sound interventions. Cortisol levels in the intervention groups declined significantly over time, with a clear group-by-time interaction, indicating that acoustic regulation not only mitigated acute noise-induced stress but also promoted self-adjustment and homeostatic recovery of the HPA axis. Persistent hypercortisolemia is known to cause hippocampal neuronal injury, impaired synaptic remodeling, and reduced learning–memory function, and noise exposure is a major external trigger (34). By reducing the frequency and amplitude of noise events, nursing interventions may have attenuated sustained stress activation, restoring physiological equilibrium from a hypervigilant to a stable state. The parallel decline in cortisol and PD incidence further supports the causal chain linking sound environment, stress, and neurocognition.

Moreover, functional outcomes at discharge improved markedly: the incidence of new functional impairment decreased from 18.0 to 1.9%. This finding suggests that optimizing the acoustic environment not only prevents acute neurological dysregulation but may also facilitate neural repair through improved sleep architecture and reduced physiological stress. Recent literature on sedation practices in critically ill children emphasizes that non-pharmacological interventions—such as family presence, music, and environmental noise reduction—play an important complementary role in maintaining comfort and stability alongside pharmacologic sedation strategies (35). These approaches collectively contribute to the improvement of neurobehavioral outcomes and overall recovery quality in the PICU.

Simultaneously, the total number of unexpected events declined significantly in the intervention groups, indicating systemic benefits extending beyond patient outcomes to staff performance. Jonescu et al. found that when ICU noise levels were maintained below 60 dB, nurses’ psychological fatigue indices decreased, and attentional focus improved (36). Combined with our findings, this suggests that sound environment interventions create a positive “environment–stress–safety” feedback loop in pediatric critical care, reducing neural burden in patients while enhancing nursing quality and operational efficiency.

Taken together, this study confirmed from acoustic, neurophysiological, and behavioral perspectives that comprehensive sound environment management is highly effective. The synergistic mechanism of external noise peak suppression and internal WN masking significantly reduced overall PD risk and facilitated early neural recovery. The protocol is simple, highly compliant, and low-cost, offering strong potential for clinical application and scalability. Future research integrating real-time acoustic monitoring, electroencephalography, and metabolic profiling may further elucidate the precise pathways of acoustic intervention, providing new evidence for non-pharmacological neuroprotection in pediatric critical care.

## 5 Limitations

This study has several limitations. First, it adopted a quasi-experimental design rather than a randomized controlled trial. Although temporal segmentation and baseline matching were used to reduce bias, potential selection confounding cannot be fully excluded. Second, the study was conducted in a single center; although the sample size met the requirements of power analysis, the generalizability of the findings warrants multicenter validation. Third, the study focused primarily on short-term neuropsychological outcomes without follow-up assessment of post-discharge cognitive function, emotional state, or long-term neurodevelopment. Future research should integrate neuroimaging and metabolic biomarkers to elucidate long-term mechanisms. Finally, variations in WN parameters—including volume, spectral composition, and individual response differences—require further exploration.

## 6 Conclusion

This study systematically verified, for the first time in a Chinese PICU setting, the effects of a comprehensive sound environment management program on PD prevention and neural functional recovery in children. The results demonstrated that the nurse-led dual intervention—noise control combined with WN masking—significantly reduced the overall incidence of PD and the proportion of hypoactive PD, while promoting reductions in physiological stress levels and improvements in neurological function. The intervention mitigated acoustic fluctuations through a “peak-trimming and valley-filling” strategy and stabilized auditory perception and arousal thresholds via WN masking, thereby interrupting the pathological “noise–stress–delirium” cascade. This cooperative mechanism of external sound source control and internal perceptual regulation highlights a new nursing model integrating environmental and sensory modulation. The intervention was simple to implement, low-cost, and highly acceptable, demonstrating strong potential for clinical dissemination.

## Data Availability

The raw data supporting the conclusions of this article will be made available by the authors, without undue reservation.
