# Supplementary material for: Application of a comprehensive sound environment management program to reduce the incidence of delirium in a pediatric intensive care unit: a quasi-experimental study
Source: Front Med (Lausanne). 2025 Dec 2;12:1721666. doi: 10.3389/fmed.2025.1721666 (PMC12705631; doi:10.3389/fmed.2025.1721666)
Supplement: Supplementary file 1 [file Table_1.docx]

**Supplementary materials**

**1. Grouping and Interventions**

**1.1 Control Group — Routine PICU Care (Group A)**

Participants admitted between October 2022 and May 2023 who met the inclusion criteria received standard PICU care, which included the following components:

(1) **Implementation of medical orders and basic nursing care:** All treatments were performed according to physicians’ prescriptions. Comprehensive basic nursing was provided, including morning and evening hygiene care, oral care, and perineal care, to maintain cleanliness and comfort.

(2) **Hourly monitoring and documentation of vital signs:** Patients’ heart rate, blood pressure, respiratory rate, and level of consciousness were monitored and recorded hourly to enable timely identification and management of any clinical deterioration.

(3) **Regular ward rounds and prevention of complications:** Nurses conducted hourly ward rounds to ensure secure fixation of all tubes (e.g., urinary catheter, gastric tube, drainage tube) to prevent dislodgement or infection. Patients were repositioned regularly to prevent pressure injuries and other complications.

(4) **Implementation of specialized nursing care:** According to each patient’s condition, specialized care measures were provided, including airway management, central venous catheter care, drainage management, and postural nursing, to ensure the effectiveness of medical treatment and promote recovery.

(5) **Environmental adjustment to promote sleep:** Curtains were adjusted according to circadian rhythm to create a quiet and comfortable sleep environment, thereby improving sleep quality and facilitating recovery.

(6) **Delirium monitoring and management:** The responsible nurse assessed PD twice daily (08:00 and 20:00) using the Cornell Assessment of Pediatric Delirium (CAPD). Any change in consciousness triggered immediate reassessment. If PD was confirmed, the physician was notified promptly, and appropriate interventions were implemented according to medical advice to ensure patient safety and optimize outcomes.

**1.2 Intervention Group — Sound Environment Control (Group B)**

From July to November 2023, patients meeting the inclusion criteria were assigned to this group.

(1) **Establishment of the implementation team**
An implementation team was formed, composed of one associate chief physician, two attending physicians, two head nurses (including the principal investigator), and all trained bedside nurses. Their responsibilities were as follows: the associate chief physician oversaw and coordinated the intervention; the attending physicians evaluated patients’ conditions and provided clinical guidance; the head nurse organized theoretical and technical training, supervised implementation, and ensured quality control; and the bedside nurses executed interventions, collected samples, and documented data to guarantee consistency and completeness.

(2) **Standardized staff training**
Prior to implementation, all nurses received structured training led by the investigator. The content, presented via PPT, covered the intervention measures, scale application, and key precautions to ensure consistent understanding. Training was conducted twice, each session lasting 40–50 minutes, followed by distribution of reference materials. Post-training, all staff underwent on-site supervision and assessment to ensure procedural and evaluative consistency. Those failing the assessment were retrained until qualified.
The intervention team finalized standardized procedures through group discussion and developed a bedside quick-reference manual to facilitate implementation. Before each intervention phase, staff received collective briefings detailing operational requirements. Refresher training and assessments were conducted every two months. Multiple formats—including PPT lectures, instructional videos, and live demonstrations—were used to enhance engagement and effectiveness.

(3) **Implementation process of sound environment control**
On the basis of routine care, trained researchers implemented the following sound management measures:

① **Device noise management:** The alarm volume of all monitoring equipment was set between 40% and 50%, with thresholds adjusted according to patient age and condition to reduce false alarms. Devices were switched to standby when patients left the bed to avoid unnecessary noise. ECG electrodes were replaced daily during morning care to ensure signal quality. Red-light alarms were handled within 20 seconds and yellow-light alarms within 60 seconds to prevent prolonged disturbance. For mechanically ventilated patients, the “suction mode” was activated during suctioning to prevent false alarms caused by disconnection.

② **Environmental management:** Staff were required to keep mobile phones at low volume (50–70%) or vibration mode to minimize non-essential noise. Automated devices were used whenever possible to reduce manual sound generation. Visual noise monitors were installed in the unit to display real-time decibel levels and remind staff to take prompt noise-reduction measures.

③ **Behavioral modification:** Staff received regular training on noise awareness and behavioral control. During communication, they maintained a one-meter distance and kept their voice between 40–55 dB, avoiding nonclinical conversations at the bedside. Nursing procedures were performed gently, concentrated in time, and preferably outside patients’ sleep periods to minimize disturbance. Agitated or crying children were soothed promptly using appropriate comfort tools. For invasive procedures, sedation or analgesia was administered according to medical advice to reduce distress and procedure-related noise.

④ **Noise masking:** From 22:00 to 06:00 daily, patients were provided with earplugs as a noise-shielding measure. For communicative children, the purpose and procedure of earplug use were explained in advance to obtain assent. Depending on the child’s age and cooperation, earplugs were gently secured with medical adhesive tape to maintain their position during sleep. Nurses routinely checked earplug placement during hourly rounds. If earplugs were dislodged unintentionally, they were not forcibly replaced to avoid disturbing the child’s rest.

**1.3 Intervention Group — Sound Environment Control Combined with WN (Group C)**

From December 2023 to April 2024, patients who met the inclusion criteria were assigned to this group. On the basis of the sound environment control measures applied in Group B, this group additionally received WN intervention. The specific procedures were as follows:

(1) **Acquisition and content of WN:**
The investigator downloaded all WN audio files from a designated music platform ([https://y.qq.com/n/ryqq/albumDetail/002Hp5ZR1HQ8Oi](https://y.qq.com/n/ryqq/albumDetail/002Hp5ZR1HQ8Oi)). The selected sounds primarily simulated natural ambient noises, including gentle rainfall, flowing streams, waterfalls, and birdsong. These tracks were carefully screened to create a calm and soothing auditory environment suitable for pediatric patients.

(2) **Playback equipment and settings:**
WN was played using a portable MP4 music player placed approximately 10 cm from the patient’s ear on the bedside. Following the recommendations of the American Academy of Pediatrics [18], the playback volume was maintained between 40 and 55 dB, with the hourly peak sound level not exceeding 65 dB to ensure auditory safety and comfort.

(3) **Playback schedule and duration:**
WN was played three times daily—from 10:00 to 11:00 in the morning, 15:00 to 16:00 in the afternoon, and 20:00 to 21:00 in the evening—with each session lasting one hour. These time slots were chosen to avoid major nursing or treatment activities (e.g., shift handovers, medication rounds) to minimize interference with routine medical procedures. We acknowledge that future studies could consider aligning white noise exposure more closely with natural sleep onset times (e.g., nap times or bedtime) to potentially enhance its sleep-promoting effects.

(4) **Personalized selection and loop playback:**
Children capable of expressing personal preferences were allowed to select their preferred tracks from the preloaded WN playlist. For infants or patients unable to make independent choices, the device was set to continuous loop mode to ensure uninterrupted playback and adaptability to individual needs.

**2. Establishment of the implementation team**An implementation team was formed, composed of one associate chief physician, two attending physicians, two head nurses (including the principal investigator), and all trained bedside nurses. Their responsibilities were as follows: the associate chief physician oversaw and coordinated the intervention; the attending physicians evaluated patients’ conditions and provided clinical guidance; the head nurse organized theoretical and technical training, supervised implementation, and ensured quality control; and the bedside nurses executed interventions, collected samples, and documented data to guarantee consistency and completeness.

**3. Standardized staff training**Prior to implementation, all nurses received structured training led by the investigator. The content, presented via PPT, covered the intervention measures, scale application, and key precautions to ensure consistent understanding. Training was conducted twice, each session lasting 40–50 minutes, followed by distribution of reference materials. Post-training, all staff underwent on-site supervision and assessment to ensure procedural and evaluative consistency. Those failing the assessment were retrained until qualified.
The intervention team finalized standardized procedures through group discussion and developed a bedside quick-reference manual to facilitate implementation. Before each intervention phase, staff received collective briefings detailing operational requirements. Refresher training and assessments were conducted every two months. Multiple formats—including PPT lectures, instructional videos, and live demonstrations—were used to enhance engagement and effectiveness.
